# Perturbation-Expression Analysis Identifies RUNX1 as a Regulator of Human Mammary Stem Cell Differentiation

**DOI:** 10.1371/journal.pcbi.1004161

**Published:** 2015-04-20

**Authors:** Ethan S. Sokol, Sandhya Sanduja, Dexter X. Jin, Daniel H. Miller, Robert A. Mathis, Piyush B. Gupta

**Affiliations:** 1 Whitehead Institute for Biomedical Research, Cambridge, Massachusetts, United States of America; 2 Department of Biology, Massachusetts Institute of Technology, Cambridge, Massachusetts, United States of America; 3 Koch Institute for Integrative Cancer Research at MIT, Cambridge, Massachusetts, United States of America; 4 Harvard Stem Cell Institute, Cambridge, Massachusetts, United States of America; University of California, San Diego, UNITED STATES

## Abstract

The search for genes that regulate stem cell self-renewal and differentiation has been hindered by a paucity of markers that uniquely label stem cells and early progenitors. To circumvent this difficulty we have developed a method that identifies cell-state regulators without requiring any markers of differentiation, termed Perturbation-Expression Analysis of Cell States (PEACS). We have applied this marker-free approach to screen for transcription factors that regulate mammary stem cell differentiation in a 3D model of tissue morphogenesis and identified RUNX1 as a stem cell regulator. Inhibition of RUNX1 expanded bipotent stem cells and blocked their differentiation into ductal and lobular tissue rudiments. Reactivation of RUNX1 allowed exit from the bipotent state and subsequent differentiation and mammary morphogenesis. Collectively, our findings show that RUNX1 is required for mammary stem cells to exit a bipotent state, and provide a new method for discovering cell-state regulators when markers are not available.

## Author Summary

The discovery of stem cell regulators is a major goal of biological research, but progress is often limited by a lack of definitive markers capable of distinguishing stem cells from early progenitors. Even in cases where markers have been identified, they often only enrich for certain cell states and do not uniquely identify states. While useful in some contexts, such enriching markers are ineffective tools for discovering genes that regulate the transition of cells between states. We present a method for identifying these cell state regulatory genes without the need for pre-determined markers, termed Perturbation-Expression Analysis of Cell States (PEACS). PEACS uses a novel computational approach to analyze gene expression data from perturbed cellular populations, and can be applied broadly to identify regulators of stem and progenitor cell self-renewal or differentiation. Application of PEACS to mammary stem cells resulted in the identification of RUNX1 as a key regulator of exit from the bipotent state.

## Introduction

Adult stem cells are functionally defined based on their ability to regenerate tissues. This unique regenerative ability can be recapitulated in culture models, where single stem cells, but not differentiated cells, form tissue rudiments in three-dimensional extracellular matrices. These tissue rudiments, or organoids, exhibit many of the topological, functional and phenotypic traits of the corresponding tissue. For example, mammary stem cells form ducts and lobules in collagen matrices that resemble structures present in the breast [1–3], while colon stem cells form mini-crypts in Matrigel that resemble analogous structures in the small intestine [4].

Given their potential for regenerative medicine, there is significant interest in identifying genes that regulate self-renewal or differentiation of stem cells. In systems with well-defined markers of stem, progenitor and differentiated states, this can be accomplished by inhibiting candidate genes and assessing the resulting effects on cell state proportions [5]. However, for many tissues markers of stem cells and early progenitors are not available, and even in cases where such markers are available they often only enrich for states of interest. This lack of defining markers has complicated efforts to screen for cell-state regulators, because changes in the number of cells expressing an enriching marker may not quantitatively reflect changes in the stem or progenitor cell types of interest.

We have addressed this difficulty by developing a new approach that identifies cell state regulators without requiring defining markers of cell state, termed Perturbation-Expression Analysis of Cell States (PEACS). Application of PEACS to mammary stem cells led to the discovery of a novel role for RUNX1 in exit from the bipotent state. We anticipate that PEACS will be useful in the many contexts where defining markers are not available, and have implemented the algorithm as a software tool available to the scientific community.

## Results

### Perturbation-Expression Analysis of Cell States (PEACS)

The analysis underlying PEACS is based on several observations. First, populations of stem cells propagated in culture are heterogeneous, and invariably include early progenitors and other more differentiated cell types. While typically considered a drawback of maintaining stem cells in culture, this heterogeneity is essential for the computational analysis underlying PEACS. Second, experimental conditions that perturb transitions between stem and progenitor states will also perturb the relative proportions of stem and progenitor cells in a heterogeneous population of cells. For example inhibiting a gene required for stem cell self-renewal will reduce the proportion of stem cells in a heterogeneous population, with a concomitant relative increase in progenitors or other more differentiated cell types.

**Fig 1:**
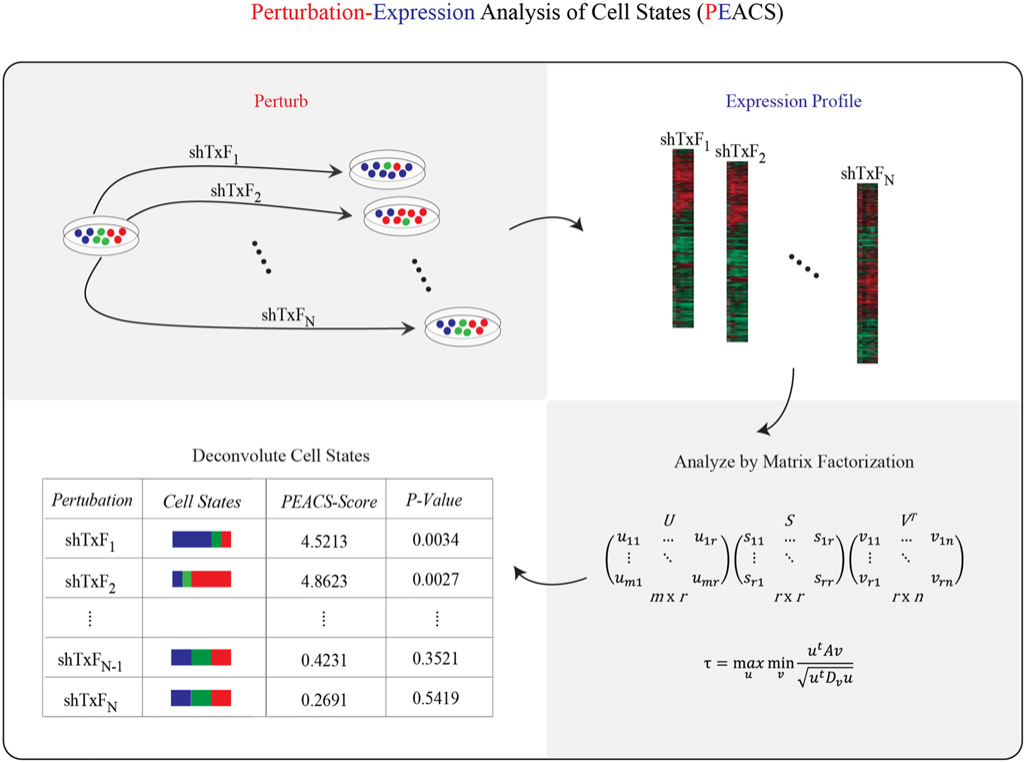
Schematic of Perturbation-Expression Analysis of Cell States (PEACS). The PEACS pipeline consists of four steps: (1) perturb a heterogeneous population with a genetic or chemical perturbation, (2) profile gene expression, (3) factor the gene-expression matrix, and (4) analyze the factored gene-expression matrix to identify perturbations that altered cell-state proportions. Shown in the lower right is the SVD formula for matrix factorization.

The computational challenge then is to use the population expression vectors—one for each perturbation—to infer which perturbations modulate cell-state proportions. However, without knowing either the cell state proportions or the gene-expression vectors of the individual states, it may appear that there is insufficient information to make such an inference. The solution lies in a third key observation: the gene-expression profiles (vectors) of heterogeneous populations of cells are weighted linear combinations of the expression profiles (vectors) of the component states within the population, with the weights in this linear combination corresponding to cell-state proportions. In other words, the gene-expression signal of the population is a *linear* mixture of component signals, the latter of which are unknown. The key is to deconvolute this signal (Fig 1).

Several computational algorithms have been designed precisely for this purpose—to infer the constituent components of mixed signals—under the assumption that the mixed signal is a weighted linear combination of constituent components. The most commonly used algorithm to infer linear components, SVD/PCA, iteratively minimizes the reconstruction error of a mixed signal, under the constraint that the component newly identified in a given iteration be orthogonal to all of the previously identified components. Given the immense success of SVD/PCA in solving many problems across diverse fields, we decided to assess its effectiveness for our problem. A second algorithm, NMF, reconstructs mixed signals by identifying components which have only non-negative loadings. Some researchers have found this non-negative constraint to be appealing, since negative loadings of genes can be difficult to interpret biologically; for this reason we also included this method for comparison. A third algorithm, ICA, does not require that the constituent components be orthogonal to one another—and instead identifies components by maximizing their independence in a statistical sense. ICA has proven useful for deconstructing mixed signals (e.g., audio) into their constituent parts.

**Fig 2:**
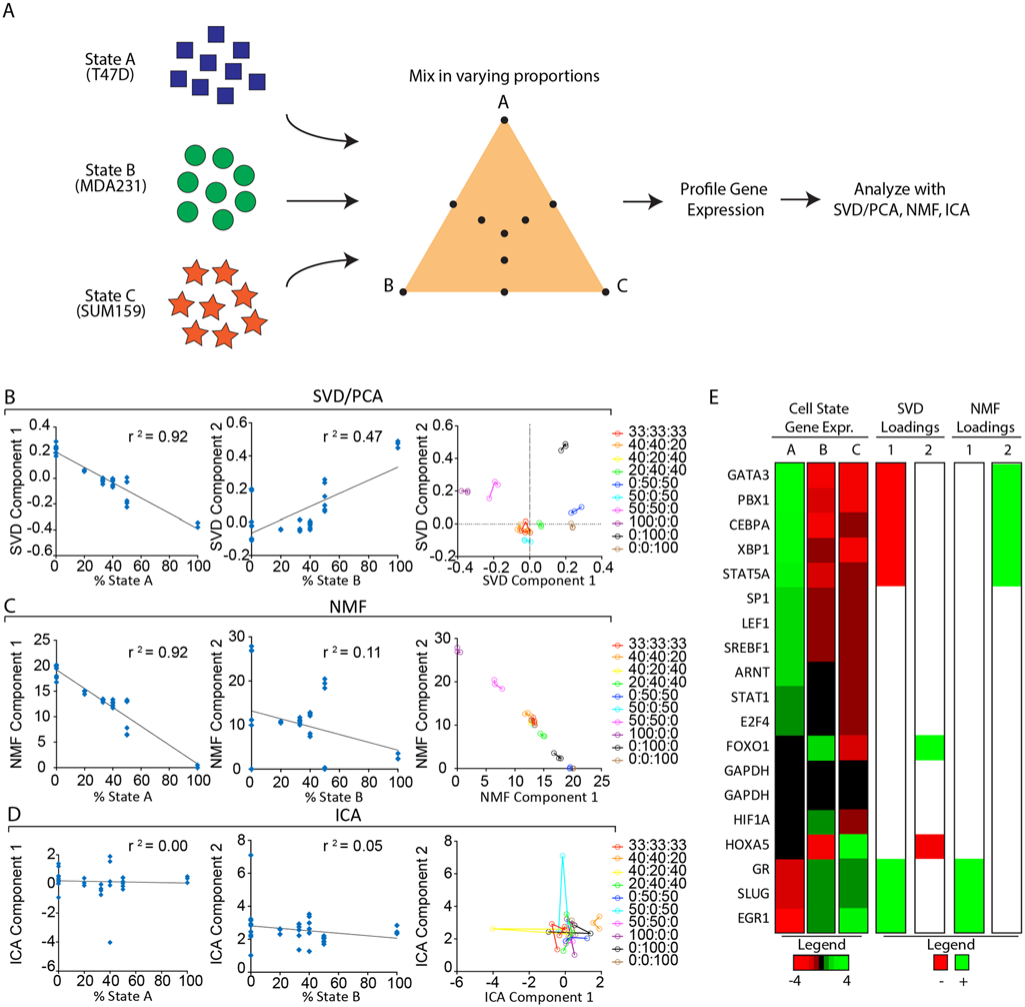
Comparison of SVD/PCA, NMF and ICA in an idealized experiment with defined cell-state proportions. (A) Three different cell lines T47D, MDA231, and SUM159 representing ‘cell states’ A, B, and C, respectively, were mixed in varying ratios. These heterogeneous mixtures were expression-profiled using qPCR, and SVD/PCA, NMF, and ICA were used to factor the resulting gene-expression matrix. For each heterogeneous population the first and second components obtained by the SVD/PCA (B), NMF (C), and ICA (D) factorization algorithms were plotted against the fraction of cells in State A or B, respectively; the squared correlation coefficient (*r* ^2^) is shown for each plot. (B-D, plot on right) For each algorithm the data were plotted in Component 1 vs Component 2 space with the replicates having the same color and connected by lines. (E) Heatmap of gene expression in each of states A, B and C. Data were log-transformed and mean-normalized by row; a red/green color scale is shown. Also shown are the genes with the highest loadings, both positive (green) and negative (red), in the first two components identified by the SVD and NMF factorizations. The heatmap shows that SVD component 1 identified genes strongly up or down in state A, while SVD component 2 found the two genes strongly differentially expressed between states B and C. In contrast, both NMF components 1 and 2 identified genes that were unique to state A, being down and up respectively in state A.

Although our goal in developing PEACS was to apply it in settings where neither the state expression vectors nor cell-state proportions are known, to assess the effectiveness of the algorithms described above (SVD, NMF, ICA) we needed an idealized context in which cell-state proportions could be experimentally defined. Experimentally defining cell-state proportions would make it possible to assess, for each algorithm, how well it identified changes in cell-state proportions across experimental conditions. To generate such idealized experimental conditions we mixed three different breast cancer cell lines (T47D, SUM159, MDA-MB-231) in defined proportions—for example 1:1:1, 1:2:2, 1:1:0—with 10 mixtures in total. In this idealized experiment the three cancer lines represented different “cell states” that were mixed in defined proportions to create heterogeneous populations (Fig 2A; T47D = State A, MDA-MB-231 = State B, SUM159 = State C). We isolated total mRNA from these heterogeneous populations and profiled the expression of 17 differentiation-related genes and GAPDH, thereby generating a gene-expression profile for each heterogeneous population (S1 Table). Lastly, we applied SVD, NMF and ICA to the gene expression matrix to assess the relative performance of these algorithms in identifying changes in cell-state proportions.

The results of the SVD, NMF and ICA analyses are presented in Fig 2B–2D. SVD/PCA successfully identified components that closely correlated with the proportions of the cell states in our idealized experiment: the first component exhibited a strong negative correlation with the fraction of cells within the population in State A (*r* ^*2*^ = 0.92), while the second component correlated with the fraction of cells in State B (*r* ^*2*^ = 0.47). Additionally, the replicates for each perturbation clustered closely together in the space spanned by these first two components identified by the SVD/PCA algorithm (Fig 2B *right*). Moreover, the first two SVD components together explained ~90% of the variation in the gene-expression data (as can be seen by the Scree plot in S1A Fig), which is consistent with the two degrees of freedom inherent in the design of this idealized experiment. In contrast to SVD/PCA, the two components identified by NMF both correlated strongly with the fraction of cells in State A (*r* ^*2*^ = 0.92 and 0.92 respectively)—with component 1 correlating negatively with the proportion of cells in State A, and component 2 correlating positively with the proportion of cells in State A (Fig 2C, S1E Fig); neither NMF component 1 nor 2 was correlated with states B or C (all *r* ^*2*^ < 0.43; S1E Fig). For this analysis the NMF factorization was performed with parameter k = 2, because the two components together explained over 95% of the variance in the gene-expression data (S1B Fig). As was the case for the SVD/PCA algorithm, the replicates for each perturbation clustered closely in the space spanned by the two components identified by NMF; this strongly suggested that the components identified by the algorithm reflected biological signal rather than experimental noise. Unlike the SVD/PCA and NMF algorithms, the first two ICA components did not correlate with the fraction of cells in any of states A, B or C (all pairwise *r* ^*2*^ < 0.13, Fig 2D, S1F Fig). Moreover, in almost all cases the various replicates for a given perturbation did not cluster together in the space spanned by the first two components identified by ICA (Fig 2D *right*).

Collectively these observations indicated that both the SVD/PCA and NMF algorithms effectively identified components that correlated strongly with cell-state proportions, while ICA failed to do so. Moreover, these observations showed that only the SVD/PCA components spanned the 2 degrees of freedom inherent this idealized experiment, which, by design, involved cellular populations that were mixtures of exactly 3 cell states.

One potential explanation for why the SVD and NMF components tracked cell-state proportions is that the components were identifying genes differentially expressed between cell states. We could directly compare gene loadings in the various components with gene expression in the various states because the gene-expression profiles of the pure states were known in our idealized experimental conditions (Fig 2E). This comparison revealed that genes with the highest loadings in SVD component 1 were uniquely expressed or repressed in state A; this was consistent with the observation that this component tracked with the fraction of cells in state A. Similarly, NMF components 1 and 2—both of which also tracked with state A—identified a very similar set of genes uniquely expressed by state A (Fig 2E). A key difference, however, was that unlike SVD component 1, which included positive and negative loadings corresponding respectively to genes down or up in state A, both of the NMF components had only positive gene loadings—with NMF component 1 having positive gene loadings for the genes down in state A, and NMF component 2 having positive loadings for the genes up in state A (Fig 2E). In contrast, SVD component 2 identified the only two genes that were strongly differentially expressed between states B and C (HOXA5, FOXO1; Fig 2E); these two genes, HOXA5 and FOXO1, were respectively down and up in state B relative to state C, and were expressed near median levels in state A. Thus, the highest loadings of SVD1 in this idealized experiment marked genes differentially expressed between luminal and basal cells, including the established luminal markers GATA3 and STAT5A. More generally, these findings suggested that the highest loadings in the SVD component vectors may serve to identify markers of specific cell states in contexts where such markers are not known.

Since our goal in developing PEACS was to identify perturbations that affect cell state proportions, we needed a method for reducing the SVD component weights to a single score that quantifies the extent of change in cell-state proportions. For this purpose the Euclidian metric, which corresponds to the natural notion of ‘distance’ in 1, 2 and 3-dimensional space, was attractive for several reasons. First, we expect distances in SVD space to scale linearly with the extent of the change in cell state proportions. Consistent with this, analysis of the SVD1 v SVD2 replicate plot for the idealized experiment (Fig 2B right panel) revealed that small perturbations in cell state proportions (e.g. 1:1:1 to 1:2:2) resulted in small distances in component space, whereas large changes in cell state proportions (e.g. 1:1:1 to 0:1:1) resulted in large distances in SVD component space. Second, the Euclidian metric makes it straightforward to quantify how noise in the various dimensions impacts the reliability of multidimensional distance estimates.

**Fig 3:**
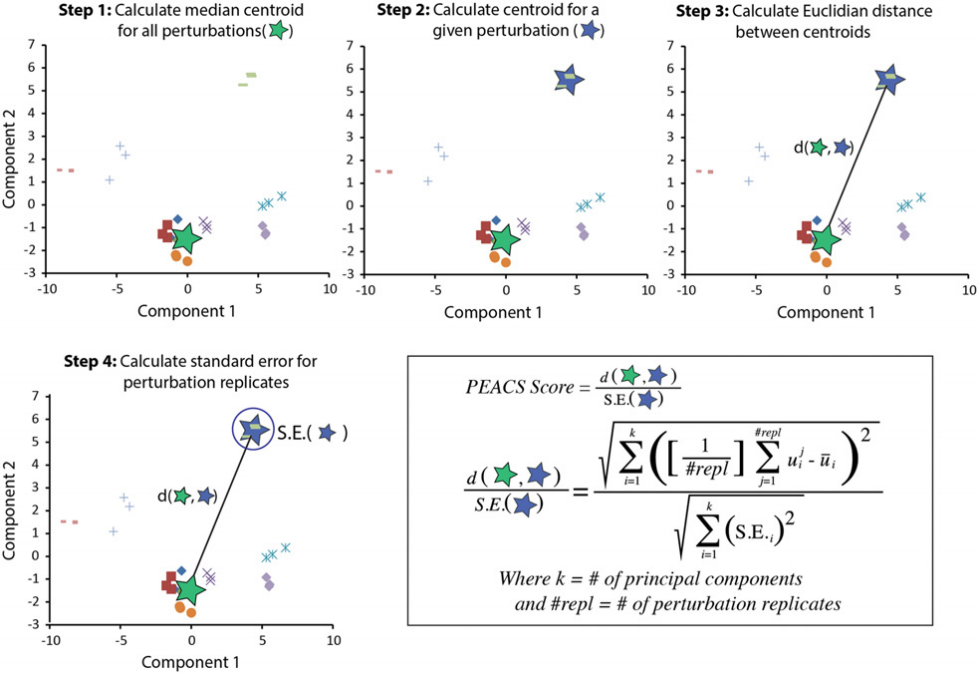
Schematic showing the steps in the calculation of a PEACS score. Step (1): calculate a median centroid for all perturbations (green star) in a given experiment. Step (2): calculate a centroid of a set of replicates for a single perturbation (blue star). Step (3): calculate the Euclidean distance (d) between the median centroid of all perturbations and the centroid of a set of replicates for a single perturbation. Step (4): calculate the standard error (S.E.) about the mean for a set of replicates for a single perturbation. For each set of perturbation replicates, the PEACS score is defined as the distance calculated in Step 3 divided by the S.E. calculated in Step 4. In the formula shown *k* is the number of SVD principal components, which is determined by a Scree plot; *repl* is the number of replicates for a given perturbation; $u_{i}^{j}$ is the coefficient of the i^th^ SVD gene-expression vector for the j^th^ perturbation; and ${\overset{-}{u}}_{i}$ is the average coefficient across all perturbation samples for the i^th^ SVD gene-expression vector.

We therefore used the Euclidean metric to compare distances between samples in the space spanned by the first k SVD components, where k was chosen using the standard approach of looking for an ‘elbow’ in the corresponding Scree plot. To account for biological variability across replicates (or different shRNAs targeting the same gene), we defined the PEACS score as the Euclidean distance divided by the standard error about the mean for each set of replicates (Fig 3).

Intuitively, this PEACS score—Euclidean distance divided by standard error—can be thought of as a ‘signal-to-noise’ ratio, which scales the magnitude of a change by the error in the distance estimate. Empirical p-values for PEACS scores were determined by Monte Carlo sampling: for a given perturbation with n replicates, a null distribution was obtained by randomly sampling n expression profiles from the experimental data, calculating a PEACS score, and iterating this process 10,000 times to generate a PEACS score null distribution. The empirical p-value was then determined by ranking the PEACS score for the given perturbation relative to the PEACS scores generated by this Monte Carlo procedure.

### Application of PEACS to a Mammary Stem Cell Model

**Fig 4:**
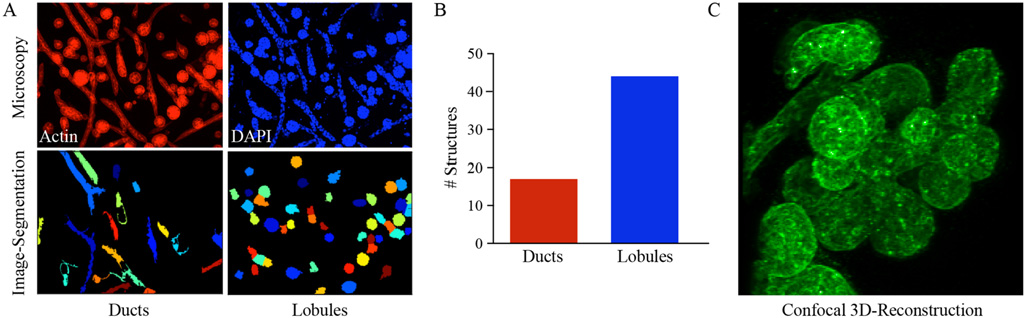
The MCF10A stem cell model exhibits multi-lineage mammary differentiation. (A, upper panels) Confocal microscopy images of MCF10A collagen cultures stained with phalloidin (red) and DAPI (blue) 8 days after seeding. (A, lower panels) The images were segmented into ductal and lobular structures using CellProfiler and quantified (B). (C) 3D confocal reconstruction of a complex ductal-lobular tissue rudiment 12 days after seeding.

We next applied PEACS to the MCF10A human stem cell model of mammary morphogenesis [6]. When seeded into a three-dimensional collagen matrix, MCF10A cells form ductal, lobular, and ductal-lobular tissue rudiments (Fig 4A–4C). These tissue rudiments are monoclonal, indicating that they arise from single stem cells, and are morphologically similar to structures present in the human mammary gland (Fig 4C; S2 and S3 Fig; S1 Movie).

As a first step, we used gene-expression profiling to identify 39 developmentally implicated transcription factors (TFs) expressed in MCF10A cells (S2 Table). We next inhibited these factors with 3–5 shRNAs targeting each TF, with two biological replicates per shRNA, resulting in a total of 240 genetically perturbed lines. For each genetically perturbed line, we then profiled the expression of all 39 factors and housekeeping genes using high-throughput qRT-PCR. These experiments generated a large data matrix with rows corresponding to gene expression values, and columns corresponding to shRNA perturbations.

**Fig 5:**
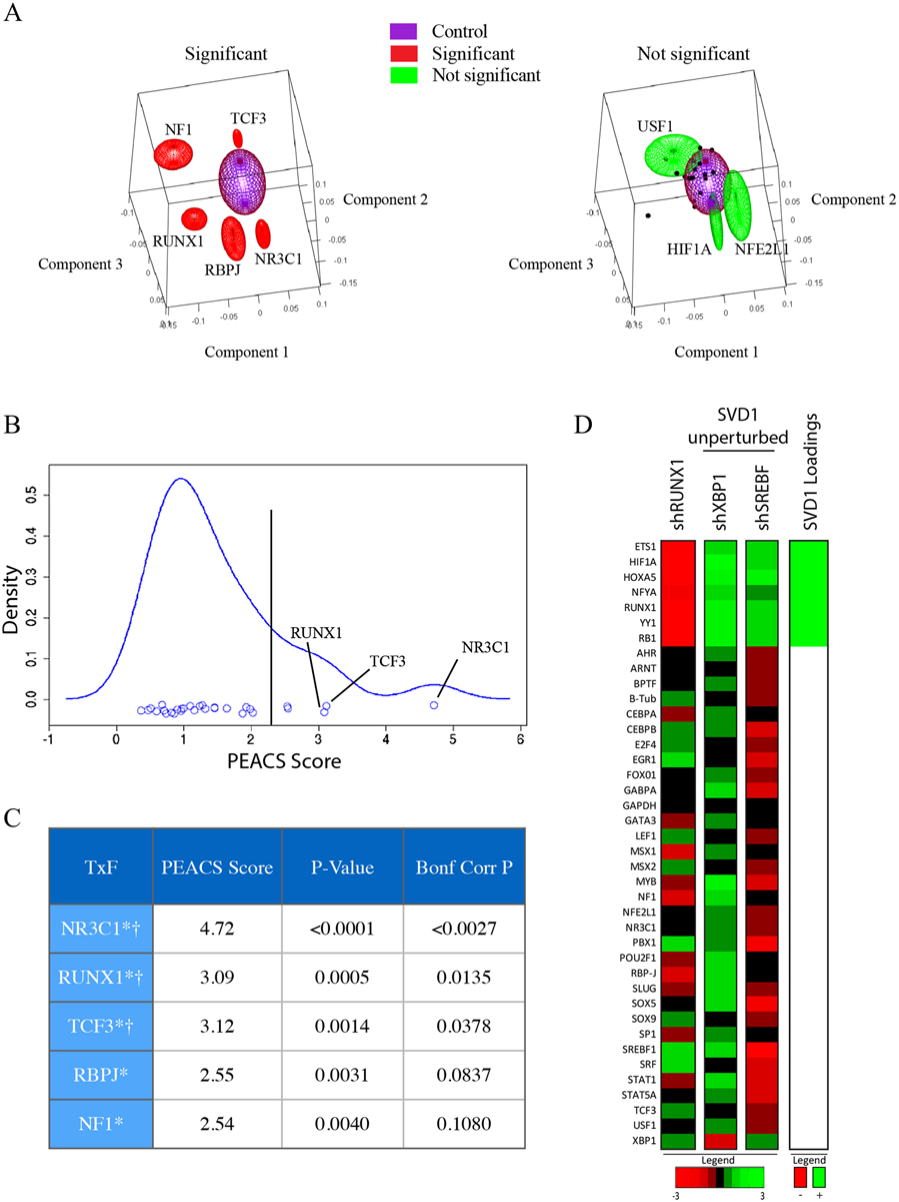
PEACS identifies RUNX1 as a candidate regulator of mammary cell state. PEACS was applied to the data matrix generated by gene-expression profiling populations of MCF10A cells perturbed by inhibiting transcription factor expression with lentiviral shRNAs. (A, left) The five genes with the highest PEACS scores are plotted as red ellipsoids centered at the coefficients for the first three SVD components, averaged across the shRNA replicates targeting the gene. The lengths of the three ellipsoid axes are the standard errors in the three respective SVD components. As a control, also shown is a purple ellipsoid centered at the first three SVD components averaged across fifteen shRNAs that failed to inhibit their target; in this case the lengths of the three ellipsoid axes are the standard deviations for the respective SVD components. (A, right) Three genes with non-significant PEACS scores (USF1, HIF1A, and NFE2L1) were plotted as green ellipsoids centered at the coefficients for the first three SVD components, averaged across the shRNA replicates targeting the gene; the three axis lengths for the ellipsoids are the standard errors in the respective SVD components. The SVD component coefficients for the remaining non-significant genes were plotted as black dots, most of which fall within the purple control ellipsoid. (B) Density estimation and (C) p-values of PEACS scores demonstrate that a few perturbations robustly affect SVD components and are statistically significant outliers. P-values were calculated by Monte Carlo resampling (see Results and Methods). † indicates Bonferroni-corrected p<0.05; * indicates nominal p<0.01 (D) Since perturbation of RUNX1 primarily affected SVD1, a heatmap was used to compare the expression profile of RUNX1-inhibited cells to the expression profile of cells in which genes predicted to have no effect on SVD1 were inhibited; in all cases the expression shown is averaged across all shRNAs targeting the given gene. The genes with the highest loadings in SVD component 1 are shown to the right of the heatmap, with strongly positive loadings shown as green, and strongly negative loadings shown as red.

From this data matrix, we eliminated genes that were not inhibited by at least 3 distinct shRNAs. Application of the PEACS algorithm to this filtered data matrix produced a score that quantified the extent to which TF inhibition affected cell-state proportions. Based on this PEACS score, most genetic perturbations had small effects on cell state proportions, which were comparable to the effects of hairpins that did not successfully knockdown their targeted genes (Fig 5A, S3 Table). When inhibited, several genes caused large, reproducible changes in cell state proportions, which could be seen when the perturbations were plotted in 3D SVD component space or as PEACS scores (Fig 5A and 5B). We used the first three SVD components for this analysis because the elbow of the Scree plot occurred at three dimensions (S1C Fig).

The top three factors identified by this analysis were NR3C1, RUNX1 and TCF3 (Fig 5C, S3 Table). Identification of the glucocorticoid receptor (NR3C1), the highest-scoring factor, was significant because of its established role in regulating mammary ductal differentiation and lactation [7]. TCF3, the third-highest scoring factor, was recently reported to be a mammary stem cell regulator [8]. RUNX1, which was the second-highest scoring factor, is mutated in a subset of breast cancers but has not been previously implicated as a regulator of mammary stem cell biology [9–11]. Since the other hits identified by PEACS were established regulators of mammary stem cells or differentiation, we suspected that RUNX1 might also play a role in one or both of these processes, and therefore decided to further explore its function.

In this dataset, RUNX1 primarily affected the expression of SVD component 1. We therefore investigated the loadings of SVD component 1 to identify the genes that have the highest contribution to this component (Fig 5D). The highest loadings of SVD component 1 were ETS1, HIF1A, HOXA5, NFYA, RUNX1, YY1, and RB1. As expected, these genes were significantly decreased in the RUNX1 knockdown condition compared to perturbation conditions that did not change SVD component 1 (Fig 5D). While we do not know what the state corresponding to SVD component 1 is, these markers may be useful for future studies investigating mammary lineages.

### RUNX1 Is Required for Mammary Stem Cells to Differentiate

**Fig 6:**
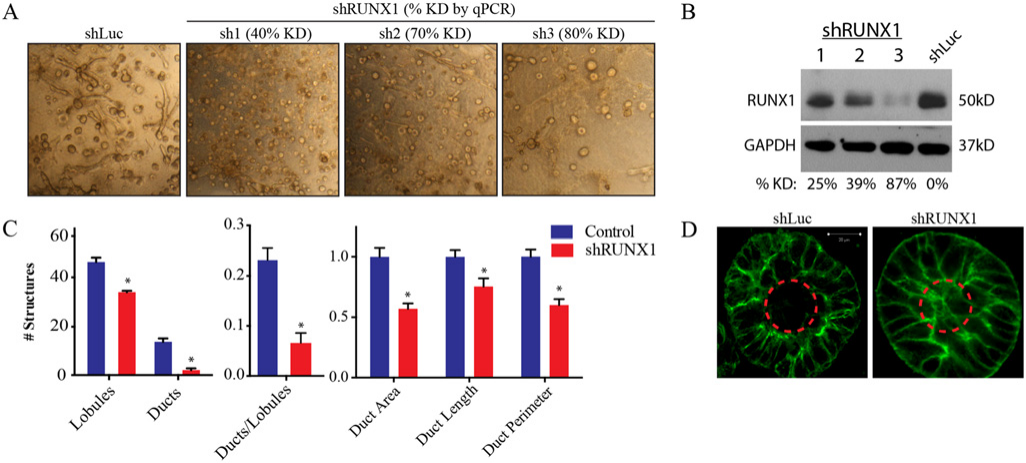
RUNX1 is required for mammary stem cells to differentiate into tissue rudiments in 3D culture. (A) Brightfield images of day 8 shRUNX1 (sh1, sh2, sh3) or control (shLuc) MCF10A cells seeded into collagen culture. % KD denotes the percent of expression lost relative to shLuc control as quantified by qPCR. (B) Western blots quantifying the percentage of expression lost relative to shLuc control at the protein level. (C) Quantification of tissue rudiments formed by shRUNX1 and shLuc cells. CellProfiler was utilized to quantify total ducts and lobules as well as ductal features (long-axis length, structure area, structure perimeter). Error bars denote SEM from 10 fields per condition. (D) Confocal microscopy of tissue rudiments of shLuc and shRUNX1 cells, taken 8 days after seeding into collagen. Dashed line indicates the hollowed region of a mature lobule.

To evaluate the functional role of RUNX1 we inhibited its expression with shRNAs (Fig 6B) and assessed the ability of MCF10A cells to form tissue rudiments in polymerized collagen. RUNX1-inhibited cells formed spheres that did not hollow (Fig 6D), indicating that they were not mature lobules, and rarely formed ducts or ductal-lobular rudiments (71% reduction relative to control); the rare ducts that did form were shorter in length (25% reduction) and did not exhibit the branched morphology seen in wild type structures (Fig 6A, 6C). As a control, cells that were either mock-infected or expressed a control shRNA were not affected in their ability to form tissue rudiments. These results indicated that RUNX1 is required for mammary cells to differentiate into ducts and mature lobules.

**Fig 7:**
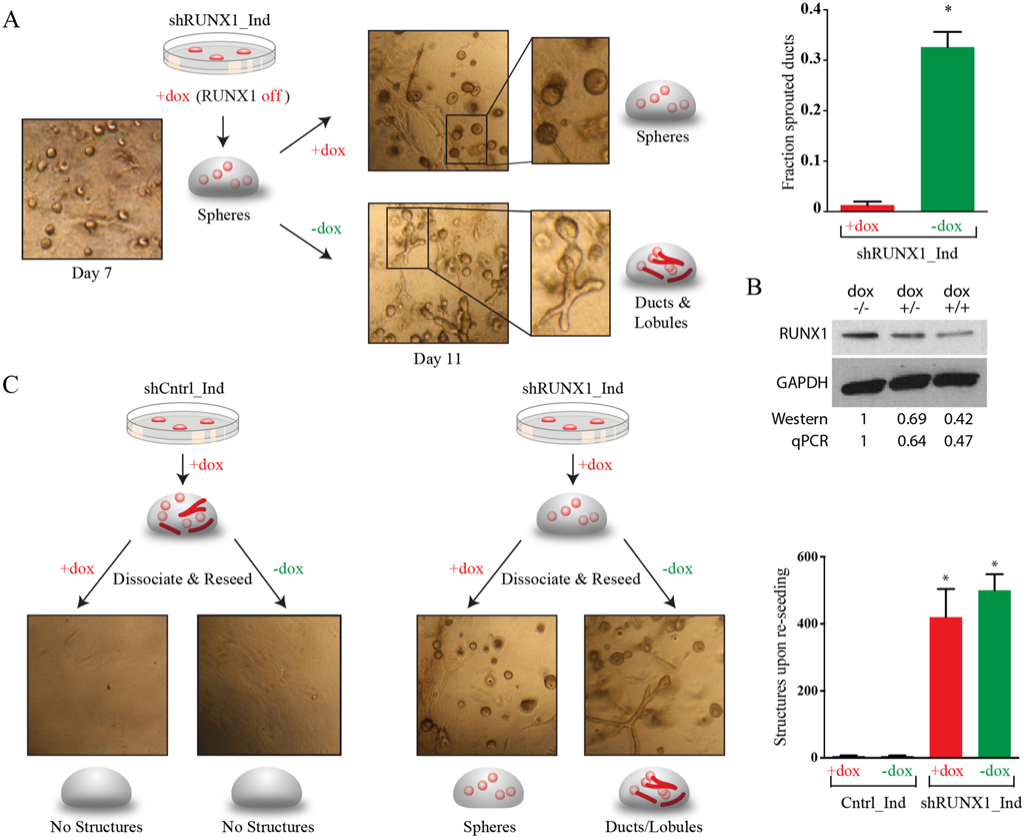
RUNX1 is necessary for mammary stem cells to exit the bipotent state. (A) Experimental schematic and brightfield images of MCF10A cells stably transduced with a dox-inducible RUNX1 shRNA, grown in collagen culture for 7 days in the presence of dox. At day 7 half of the collagen gels were removed from dox, and the other half maintained in dox for an additional 4 days. Tissue rudiments maintained in dox remained as solid spheres, whereas spheres removed from dox rapidly sprouted ducts within 12–24 hrs. (B) Western blot confirming inducible RUNX1 inhibition by the dox inducible shRNA. MCF10A cells were cultured without dox for 7 days (lane 1), or with dox for 4 days followed either by culture without dox for an additional 3 days (lane 2) or culture with dox for an additional 3 days (lane 3). Also shown is quantification of the western blot normalized to GAPDH and the no dox control treatment, and quantification using qPCR. (C) Experimental schematic, brightfield images, and quantification of MCF10A dox-inducible shRUNX1 cells that were grown in the presence of dox for seven days, removed from collagen, dissociated into single cells, and reseeded into a new collagen pad in the presence or absence of dox. While control cells are unable to reseed structures, inducible shRUNX1 cells are able to reseed structures with high efficiency (reseeding capacity shown as structures formed per 7500 cells; * indicates p<0.05 relative to wild type by t-test. SEM is indicated n = 3). Dox-inducible shRUNX1 cells grown in the absence of dox are multipotent, with the capacity to form ducts, lobules, and complex ductal-lobular structures in 13 days.

To assess if the phenotype caused by RUNX1 inhibition was reversible, we generated an MCF10A line in which RUNX1 could be reversibly inhibited by a doxycyline (dox)-inducible shRNA (Fig 7B). When cultured in collagen in the presence of dox, these MCF10A cells formed solid spheres and few ducts, recapitulating the phenotype observed above when RUNX1 was constitutively inhibited by shRNAs. When RUNX1 was re-expressed by withdrawing dox, the spheres rapidly sprouted ducts and began to hollow—often within 12–24 hours (Fig 7A). This finding indicated that the RUNX1-inhibited spheres were still capable of forming both ducts and lobules upon RUNX1 re-expression, raising the possibility that these spheres might consist of bipotent cells reversibly arrested in their differentiation.

### Inhibition of RUNX1 Traps MCF10A Mammary Stem Cells in a Bipotent State

To directly examine this possibility we assessed whether single cells from RUNX1-inhibited spheres could form tissue rudiments when seeded into collagen. Parental MCF10A cells largely lose this ability upon differentiating in collagen (Fig 7C). We seeded cells with dox to form RUNX1-inhibited spheres, harvested and dissociated the spheres by treatment with collagenase and trypsin, and then reseeded single cells into collagen with or without dox. Cells reseeded in dox again gave rise to solid spheres. However, those reseeded without dox formed lobules and ducts that matured into complex ductal-lobular structures (Fig 7C), doing so with efficiency comparable to that of parental MCF10A cells maintained in 2D culture.

These observations strongly suggested that parental MCF10A cells dissociated from tissue rudiments lost the ability to reseed tissue rudiments because they had differentiated and lost stem and progenitor activity; in contrast, cells within RUNX1-inhibited MCF10A spheres maintained their ability to reseed tissue rudiments because they did not differentiate in collagen and remained bipotent.

### Primary Human Mammary Stem Cells Require RUNX1 to Differentiate

**Fig 8:**
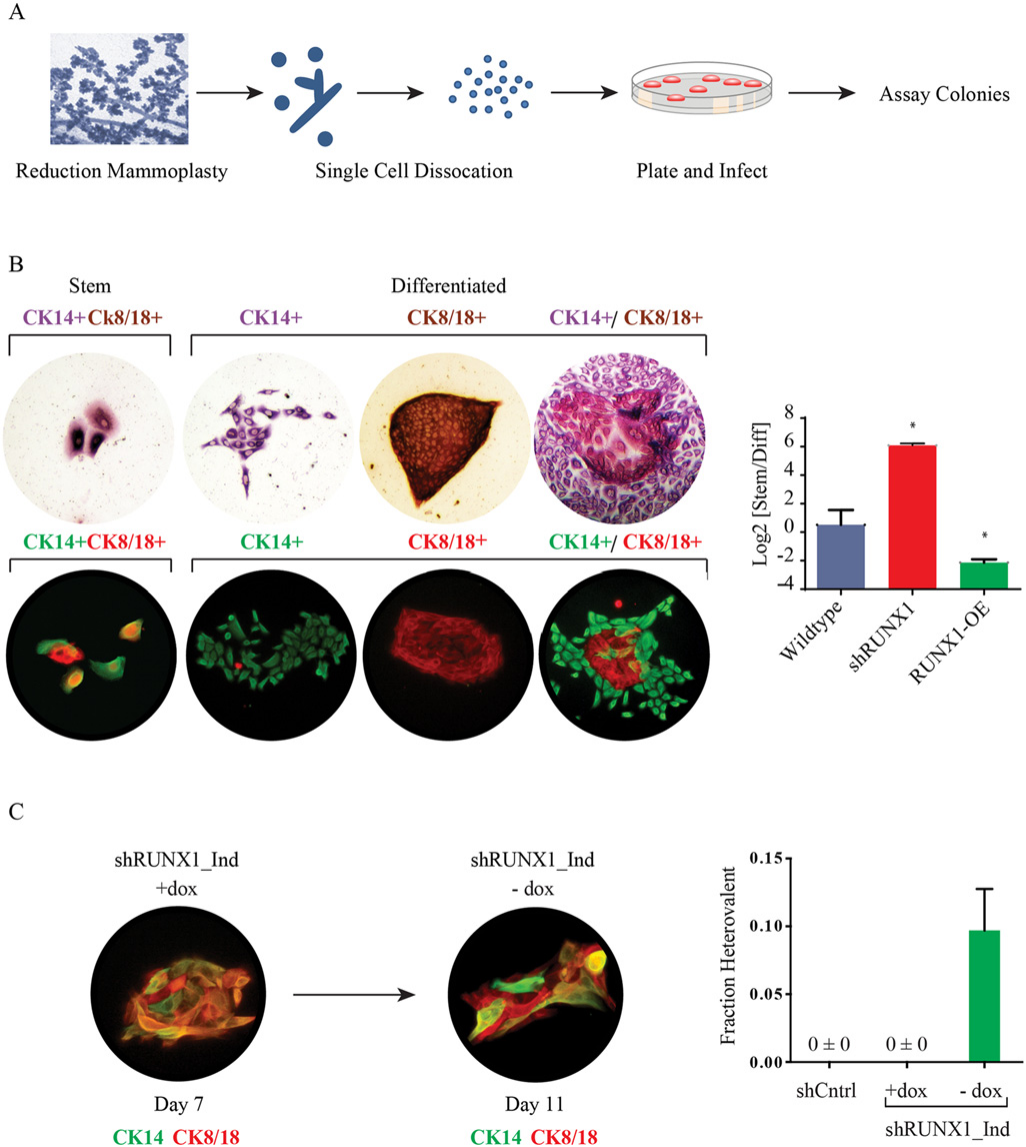
RUNX1 inhibition blocks the differentiation of patient-derived mammary stem cells. (A) Schematic of the human breast progenitor cell colony formation assay. Human primary organoids were dissociated into single cells and plated onto culture plates. (B) Brightfield and fluorescent images of CK14 and CK8/18 stained human progenitor cell colonies: stem cell colonies are small and consist of cells dual positive for CK8/18 (brown;red) and CK14 (red/purple; green); differentiated colonies are larger and have domains that are singly positive for CK8/18 and CK14. Quantification of progenitor cell colonies from control, shRUNX1 or RUNX1 overexpressing cells; (C) Immunofluorescence and quantification of heterovalent colonies formed by primary human cells infected with a dox-inducible shRUNX1 in the presence of dox for 7 days. After 7 days, dox was removed and the colonies were grown for an additional 96 hours. These cultures generated heterovalent colonies—defined as colonies containing both stem and differentiated cell types. Shown is a heterovalent colony containing both CK14,CK8/18 double-positive cells, and cells that only stained for either CK14 or CK8/18. The fraction of heterovalent colonies in each condition is shown. * indicates p<0.05 versus wild type; error bars are SEM with n = 4.

We next examined if RUNX1 also affected the differentiation of primary human breast stem and progenitor cells. To this end we isolated primary human breast epithelial cells from reduction mammoplasty tissue samples, modulated RUNX1 expression, and assessed stem and progenitor cells using colony forming assays (Fig 8A) [12–14]. In these assays the majority of stem and progenitor cells form colonies containing differentiated luminal or basal cells. However a fraction of bipotent stem cells proliferate but do not differentiate; these form micro-colonies of 2–16 cells that remain uncommitted and co-express both luminal and basal markers.

Inhibiting RUNX1 expression caused a 2-fold increase in the number of stem cell micro-colonies, suggesting that this transcription factor was required for primary human breast stem cells to differentiate in culture (Fig 8B). Consistent with this interpretation, inhibiting RUNX1 expression reduced the number of differentiated colonies by nearly 90%, while its over-expression led to a 300% increase in differentiated colonies.

We next examined whether transiently inhibiting RUNX1 would expand the population of functional stem cells in culture. For this experiment we first infected primary cells with the dox-inducible shRUNX1 lentivirus, and plated cells with dox to assay for colony-forming ability. After micro-colonies of stem cells had formed (7 days after plating), we removed the dox so that RUNX1 would be re-expressed. We found that re-expressing RUNX1 caused the stem cell micro-colonies to differentiate within 48–96 hours, and resulted in the formation of heterovalent colonies that included both bipotent stem cells and lineage-committed basal and luminal cells (Fig 8C). These heterovalent colonies were never observed in colony-forming assays with control primary cells, or in assays with primary cells in which RUNX1 had been stably inhibited.

Collectively, these findings indicate that RUNX1 inhibition enables primary breast stem cells to expand in an uncommitted state while retaining the functional ability to differentiate in culture.

## Discussion

We have shown that PEACS identifies perturbations that affect cell-state transitions, taking as input the gene-expression profiles of perturbed cellular populations. We validated PEACS by applying it to a mammary stem cell model with shRNAs as a source of perturbations. In this context, the method identified several established regulators (e.g., NR3C1 and TCF3) of mammary stem cell biology, as well as a novel gene, RUNX1, which had not previously been implicated as a mammary stem cell regulator. Follow-up studies revealed that inhibiting RUNX1 prevented mammary stem cells from differentiating, indicating that this gene is required for stem cells to exit a bipotent state. Although our study focused on shRNA perturbations, there is every reason to believe that PEACS would be equally effective for gene over-expression or chemical perturbations.

Several computational methods for analyzing gene-expression profiles have been previously reported [15–19]. PEACS differs from these in three important ways. First, the goal of PEACS is to specifically identify perturbations that influence how cells transition between differentiation states; we are not aware of other methods that do this. Second, the method does not require any markers of stem, progenitor or differentiated states. Third, our method analyzes bulk populations of cells to identify changes in cell state ratios, rather than analyzing large numbers of single cells.

We anticipate that this marker-free approach will be particularly useful in the many contexts where stem, progenitor, and differentiated cells have been identified functionally, but where markers that distinguish these states are not yet available. It is worth emphasizing that, although markers that enrich for stem and progenitor states have been identified in many systems, few systems offer markers that sort stem or progenitor cells to purity; this latter ability is essential if these markers are to be used to identify genes that regulate state transitions. In cases where such markers are in fact available—or when they are used to define states *de facto* without consideration of the underlying biology—we have previously shown that a Markov model can be used to quantify the rates of transition between states, and predict the equilibrium proportions of cell states [20].

Targeting RUNX1 may offer unique possibilities for therapeutic applications. Stem cells have a strong tendency to differentiate when propagated in culture, even under conditions that are intended to maintain them in an undifferentiated state. This problem has been observed with human ES cells, HSCs, and many other stem cell types. We have shown that primary human mammary stem cells can be expanded in a bipotent state by transiently inhibiting RUNX1; moreover these cells spontaneously differentiate once RUNX1 expression is re-established. A chemical compound that inhibits RUNX1 could therefore be used to propagate mammary stem cells in culture. It will be of interest to examine if inhibiting RUNX1 can also prevent other types of stem cells from exiting a bi- or multipotent state. In support of this possibility, a dominant-negative RUNX1 translocation has been found in a subset of leukemias, and expression of this protein blocks the differentiation of leukemic cells and promotes the self-renewal of hematopoietic stem cells [21,22]. Additionally, a RUNX1 ortholog, Runt, has been shown in planaria to be required for neoblast stem cells to differentiate at wound sites [23]. Taken together, these observations suggest the intriguing possibility that this function of RUNX1/Runt is conserved across species and cell types.

## Methods

### Ethics Statement

Primary tissues were obtained with consent in compliance with laws and institutional guidelines, as approved by the Institutional Review Board of Maine Medical Center. Exemption status for human research was obtained from the Committee on the use of Humans as Experimental Subjects (COUHES) at MIT, based on de-identification of the samples. All patient samples are de-identified prior to distribution for research use. The data collected and stored is limited to basic demographic data, specimen handling information (ex: related to chain of custody), specimen quality data, and histopathologic data. At no time is any patient identifier provided to any researcher.

### Primary Cells, Cell Lines, Tissue Culture, and Lentiviral Production

MCF10A cells were obtained from ATCC and cultured in MEGM with 100 ng/ml cholera toxin, GlutaMax, Penicillin and Streptomycin (Lonza CC-3150). HEK293T cells were maintained in DMEM supplemented with 10% FBS, GlutaMax, Penicillin and Streptomycin. SUM159 (Asterland), MDA-MB-231 (ATCC), and T47D (ATCC) cells were cultured in DMEM with 10% FBS, GlutaMax, Penicillin and Streptomycin.

Human organoids were isolated from breast tissues from patients undergoing elective reduction mammoplasty. Primary tissues were obtained with consent in compliance with laws and institutional guidelines, as approved by the Institutional Review Board of Maine Medical Center. Organoids were aliquoted in 1:1 DMEM/Hams-F12 media supplemented with 5% calf serum, 10 ng/mL insulin, 10 μg/mL epidermal growth factor, 10 μg/mL hydrocortisone, and 10% DMSO and stored in liquid nitrogen. Doxycycline (dox), where applicable, was used at a concentration of 4μg/ml.

Lentivirus production, target cell infection, and selection were performed as previously described [24]. Constitutive shRNA plasmids in a pLKO.1 vector were obtained from the Broad Institute RNAi consortium (https://www.broadinstitute.org/rnai/trc3), and inducible hairpins (dox “ON”; pTRIPZ vector) were obtained from Thermoscientific. Overexpression constructs were obtained through gateway cloning of the appropriate ORF into the pLenti6.2-ccdB-3xFLAG-V5 construct.

### PEACS: Perturbations

MCF10A cells were seeded onto a 96 well plate at a density of 7500 cells per well and infected the next day with hairpin lentivirus targeting an expressed developmental transcription factor. One day after infection, cells were selected with 5ug/ml puromycin containing media. Two days later RNA was collected with the Qiagen RNeasy 96 Biorobot 8000 kit and cDNA synthesized with the iScript cDNA synthesis kit (BioRad 170–8890).

### PEACS: Expression Profiling by qPCR

Microfluidic qPCR was carried out according to the manufacturer’s Protocol (Protocol 37: Fast Gene Expression Analysis Using EvaGreen on the BioMark or BioMark HD System). The 39 TFs profiled were selected by profiling gene-expression in MFC10As and selecting all TFs implicated in differentiation that were confirmed to be expressed by qPCR. The cDNA was preamplified for 14 cycles with a mix of 41 primer sets (39 TFs, BTub, and GAPDH) and mastermix, then treated with ExoI. Prior to analysis with PEACS, the data matrix with the Fluidigm CT values was normalized to GAPDH and median normalized by gene such that the median CT value for each gene was 0. For the idealized experiment, gene expression was profiled using standard qPCR and the 17 genes profiled were randomly selected transcription factors expressed by MCF10A cells and implicated in differentiation.

### PEACS: Algorithm

Let M be a data matrix of perturbation-expression values with rows corresponding to perturbations and columns corresponding to the genes whose expression was profiled. We used the reduced singular value decomposition to transform M, so that

$$M=\;\overset{m\;x\;n}{\overset{︷}{\left[\begin{array}{ccc} \begin{array}{c} ↑\\ u_{1}^{\;}\\ ↓ \end{array} & \begin{array}{c} \;\\ \ldots \\ \; \end{array} & \begin{array}{c} ↑\\ u_{n}^{\;}\\ ↓ \end{array} \end{array}\right]}}\;\overset{n\;x\;n}{\overset{︷}{\left[\begin{array}{ccc} \begin{array}{c} \sigma_{1}\\ \;\\ \; \end{array} & \begin{array}{c} \;\\ \ddots \\ \; \end{array} & \begin{array}{c} \;\\ \;\\ \sigma_{n} \end{array} \end{array}\right]}}\overset{n\;x\;n}{\overset{︷}{\left[\begin{array}{ccc} \leftarrow & v_{1}^{t} & \to \\ \; & \vdots & \;\\ \leftarrow & v_{n}^{t} & \to \end{array}\right]}}=\;\overset{n}{\underset{i=1}{\sum }}\sigma_{i}u_{i}v_{i}^{t}\;$$ (1)

where *u*_1_, …, *u*_*n*_ and *v*_1_,…, *v*_n_ are respectively the left and right eigenvectors corresponding to the singular values σ_i_ of the reduced singular value decomposition of M. We have assumed here that n < m, i.e., the number of perturbations exceeds the number of genes whose expression is profiled. Because the dimensions of *u*_*i*_ and *v*_*i*_ are (*mx1*) and (*nx1*), respectively, and the *σ*_*i*_ are scalars, M can be viewed as a weighted sum of the rank-one matrices $u_{i}v_{i}^{t}$.

We then use the first k singular values and vectors to reconstruct a low-rank approximation of M:

$$M\;\sim \;\;\overset{m\;x\;k}{\overset{︷}{\left[\begin{array}{ccc} \begin{array}{c} ↑\\ u_{1}^{\;}\\ ↓ \end{array} & \begin{array}{c} \;\\ \ldots \\ \; \end{array} & \begin{array}{c} ↑\\ u_{k}^{\;}\\ ↓ \end{array} \end{array}\right]}}\overset{k\;x\;k}{\overset{︷}{\left[\begin{array}{ccc} \begin{array}{c} \sigma_{1}\\ \;\\ \; \end{array} & \begin{array}{c} \;\\ \ddots \\ \; \end{array} & \begin{array}{c} \;\\ \;\\ \sigma_{k} \end{array} \end{array}\right]}}\overset{k\;x\;n}{\overset{︷}{\left[\begin{array}{ccc} \leftarrow & v_{1}^{t} & \to \\ \; & \vdots & \;\\ \leftarrow & v_{k}^{t} & \to \end{array}\right]}}$$ (2)

The value k is chosen using a Scree plot, as described in the main text. In the case of k = 3:

$$\begin{array}{c} if\;\;k=3,\;\;\;M\;\;\sim \;\;\overset{m\;x\;3}{\overset{︷}{\left[\begin{array}{ccc} \begin{array}{c} ↑\\ u_{1}^{\;}\\ ↓ \end{array} & \begin{array}{c} ↑\\ u_{2}^{\;}\\ ↓ \end{array} & \begin{array}{c} ↑\\ u_{3}^{\;}\\ ↓ \end{array} \end{array}\right]}}\overset{3\;x\;3}{\overset{︷}{\left[\begin{array}{ccc} \begin{array}{c} \sigma_{1}\\ \;\\ \; \end{array} & \begin{array}{c} \;\\ \sigma_{2}\\ \; \end{array} & \begin{array}{c} \;\\ \;\\ \sigma_{3} \end{array} \end{array}\right]}}\overset{3\;x\;n}{\overset{︷}{\left[\begin{array}{ccc} \leftarrow & v_{1}^{t} & \to \\ \;\leftarrow & v_{2}^{t} & \;\to \\ \leftarrow & v_{3}^{t} & \to \end{array}\right]}}\\ =\;\;\overset{m\;x\;3}{\overset{︷}{\left[\begin{array}{ccc} \begin{array}{c} ↑\\ u_{1}^{\;}\\ ↓ \end{array} & \begin{array}{c} ↑\\ u_{2}^{\;}\\ ↓ \end{array} & \begin{array}{c} ↑\\ u_{3}^{\;}\\ ↓ \end{array} \end{array}\right]}}\overset{3\;x\;n}{\overset{︷}{\left[\begin{array}{ccc} \leftarrow & \sigma_{1}v_{1}^{t} & \to \\ \;\leftarrow & \sigma_{2}v_{2}^{t} & \;\to \\ \leftarrow & \sigma_{3}v_{3}^{t} & \to \end{array}\right]}}\\ =\;\;\overset{m\;x\;3}{\overset{︷}{\left[\begin{array}{ccc} u_{1}^{1} & u_{2}^{1} & u_{3}^{1}\\ u_{1}^{2} & u_{2}^{2} & u_{3}^{2}\\ \vdots & \vdots & \vdots \\ \vdots & \vdots & \vdots \\ u_{1}^{m} & u_{2}^{m} & u_{3}^{m} \end{array}\right]}}\overset{3\;x\;n}{\overset{︷}{\left[\begin{array}{ccc} \leftarrow & \sigma_{1}v_{1}^{t} & \to \\ \;\leftarrow & \sigma_{2}v_{2}^{t} & \;\to \\ \leftarrow & \sigma_{3}v_{3}^{t} & \to \end{array}\right]}} \end{array}$$ (3)

The gene-expression vector for the perturbation p can therefore be approximated by the following weighted sum of the first 3 SVD eigenvectors:

$$\left[p^{th}\;row\;of\;M\right]\;\sim \;\sigma_{1}\cdot u_{1}^{p}\cdot {\vec{v}}_{1}^{t}+\sigma_{2}\cdot u_{2}^{p}\cdot {\vec{v}}_{2}^{t}+\sigma_{3}\cdot u_{3}^{p}\cdot {\vec{v}}_{3}^{t}\;,\;$$ (4)

Thus the gene expression data for each perturbation p is mapped into the space spanned by linear combinations of the first k gene-expression SVD eigenvectors *v*_1_,…, *v*_k_. Again for k = 3:

$$Expression\;data\;for\;perturbation\;p\;\to \;\left(u_{1}^{p},\;u_{2}^{p},\;u_{3}^{p}\;\right)\;$$ (5)

These coordinates in SVD-space are plotted as ‘Component scores’ in Figs 2B, 3, and 5A.

Finally, to determine the PEACS score, we first calculate the Euclidean distance between the $\left(u_{1}^{p},\;u_{2}^{p},\;u_{3}^{p}\right)$ for a given perturbation p (averaged across all its replicates), and the median centroid vector $\left({\bar{u}}_{1},\;{\bar{u}}_{2},{\bar{u}}_{3}\right)$ taken across all m perturbations:

$$d\left(gene\;perturbed,\;centroid\;all\;perts\right)=\;\sqrt{\sum_{i=1}^{k}{\left(\left[\frac{1}{\#repl}\right]\sum_{\#repl}^{j=1}u_{i}^{j}-\;{\bar{u}}_{i}\right)}^{2}}$$ (6)

Finally, the PEACS score is calculated by dividing the distance in (Eq 6) by the standard error across replicates for a given perturbation.

$$PEACS\;Score\left(gene\;perturbed\right)=\;\frac{\sqrt{\sum_{i=1}^{k}{\left(\left[\frac{1}{\#repl}\right]\sum_{j=1}^{\#repl}u_{i}^{j}-\;{\bar{u}}_{i}\right)}^{2}}}{\sqrt{\sum_{i=1}^{k}{\left(S.E._{i}\right)}^{2}}}$$ (7)

To calculate a p-value, a Monte Carlo sampling algorithm was implemented. For each set of n perturbations, a null distribution of PEACS scores was obtained by sampling n random perturbations 10,000 times without regards for perturbation labels. The p-value was defined as the rank of the real PEACS score in the null distribution divided by 10,000.

The PEACS code for MATLAB is available as a supplemental file (S2 Text) and on our lab website at: http://guptalab.wi.mit.edu/.

### Collagen Culture

7.5x10^3^ MCF10A cells were resuspended in 0.2ml of collagen solution (1.25mg/ml rat tail collagen I in PBS, brought to pH 7.3 with 0.1N NaOH) and plated on a single chamber of a 4-chamber slide. Collagen was polymerized for 2 hours at 37°C, after which they were detached and cultured in 1ml of MCF10A medium.

### Reseeding Assay

MCF10A cells were grown in collagen matrix through day 7, at which time the collagen pads were collected and incubated in 100 ug/ml collagenase in PBS at 37°C for 10 minutes. The structures were collected by centrifugation (500 RPM, 5 min), resuspended in 0.25% trypsin, and incubated for 20–25 minutes at 37°C. Cells were counted in trypan blue, spun down (500RPM, 5 min), and resuspended in MCF10A media; 7500 living cells were reseeded into a new collagen pad.

### Immunofluorescence

Samples were fixed with 4% paraformaldehyde for 15 minutes at room temperature. Pads were permeabilized using 0.1% TritonX-100 and incubated with blocking solution (PBST with 10% goat serum and 3% BSA) for 1 hr at room temperature and stained with the appropriate primary antibody in blocking buffer for 1–2 hours at room temperature or overnight at 4°C. The samples were washed with PBS, and incubated with an Alexa Fluor-labeled secondary antibody. Samples were washed, stained with 1ug/ml DAPI. Images of phalloidin-AF594 and DAPI- stained collagen structures were analyzed by image segmentation software (CellProfiler; [25]), with an analysis pipeline that differentially detected lobules and ducts based on size, area and form factor adjustments.

### Colony Assay

Primary human organoids were thawed and plated on a 10cm dish in 10ml of RMFC (DMEM + 10% Calf Serum) media for 1–2 hours. The non-adherent fraction, fibroblast reduced organoids, was collected, spun 10 minutes at 233 gravity, resuspended in cold PBS and passed 10 times through an 18-gauge needle. The organoids were once again pelleted 5 minutes at 335 gravity, resuspended in 2ml of 0.05% trypsin, and incubated 10 minutes at 37°C. We then added 8ml of RMFC media and 0.5mg of DNaseI (Roche 10104159001). The cell suspension was passed through a 40 um filter and the cells counted. Thirty thousand cells were plated per well of a 6-well plate in MEGM, and assayed for cytokeratin expression after 7–11 days, using CK8/18 antibody (Vector VP-C407) and CK14 antibody (Thermo 9020-P). Some plates were visualized using IHC, while others were visualized using IF with AF488/AF555 conjugated secondary antibodies.

### Immunohistochemistry/Western Blot

Colonies grown on 6-well plates were fixed in 100% methanol for 5 minutes, washed with PBS, permeabilized with 0.1% triton X-100 followed by serial blocking in 3% hydrogen peroxide and 1% BSA + 2% horse serum. The plates were incubated overnight at 4°C with 1:750 CK8/18 antibody. The plates were incubated with 1:200 αMouse-IgG-HRP (Vector BA-2000) for 30 minutes, and stained with DAB according to the manufacturer’s protocol (Vector ABC elite PK-6100; Vector ImmPACT DAB SK-4105). Excess avidin/biotin was blocked with the Vector Avidin/Biotin blocking kit SP-2001. Plates were re-blocked for 1 hour in PBS + 1% BSA and 2% goat serum, then incubated for 1 hour at room temperature with 1:750 CK14 antibody in PBS + 1% BSA then incubated at room temperature with αRabbit-IgG-HRP (Vector BA-1000) for one hour. The plates were then stained with VIP according to the manufacturer’s protocol (Vector ABC elite PK-6100; Vector ImmPACT VIP SK-4605), washed with water and stored dry. Western blots were performed with standard procedures. RUNX1 was blotted with 1:1000 Ab23980 (AbCAM).

## Supporting Information

S1 Text Document containing the description of how the MCF10A organoids were analyzed by immunofluorescence, and how clonality was determined. (DOCX)

S2 Text PEACS code for MATLAB 2014. (TXT)

S1 Table Genes probed by qPCR in the idealized experiment referenced in Fig 2. (DOCX)

S2 Table Developmental transcription factors expressed in MCF10A cells that were targeted with shRNAs. (DOCX)

S3 Table PEACS output from MCF10A perturbations. Displayed are the PEACS scores, uncorrected p-value, Bonferroni corrected p-value, and significance (* = raw p<0.01; † = Bonferroni-corrected p<0.05) for genes with at least 3 knockdown conditions with 2-fold or higher knockdown. The negative control sets were generated by taking three random sets of 5 hairpins where the targeted gene was not successfully knocked down. P-values were obtained through Monte Carlo resampling of the PEACS scores, as described in the text.
(DOCX)

S1 Fig SVD, NMF, and ICA results. Scree plots and explained variance plots were used to decide on dimensions for SVD and NMF, respectively. These results are displayed as scatter plots where (A) the x-axis contains the SVD number and the y-axis denotes the variance explained by each SVD in the ideal experiment or (B) the x-axis contains the rank used by the NMF algorithm and the y-axis shows the fraction explained by all the components of the factorization in the ideal experiment. Similarly, (C) A scree plot of the SVD results from the MCF10A experiment was plotted to decide on dimensionality, where axes are as noted in (A). The results of first and second dimensions of (D) SVD, (E) NMF, and (F) ICA deconvolution were plotted against fractions of state A, B, or C.
(DOCX)

S2 Fig MCF10A tissue rudiments express mammary gland markers. Day 8 collagen cultures were stained for basal marker (CK14) and luminal markers (CK8/18, MUC1 and CSN2). Nuclei were stained with DAPI. Scale bar, 20 μm.
(DOCX)

S3 Fig MCF10A tissue rudiments are monoclonally derived. MCF10A cells infected with a pool of red, green, and blue viruses were seeded into collagen matrix. The structures were visualized in the red, green, and blue channel (overlay shown) at 2 (A) and 6 days (B), revealing monoclonal lobules and monoclonal ducts with occasional fusions. Images were acquired at 10X magnification.
(DOCX)

S1 Movie Panning reconstruction of the complex phalloidin stained ductal-lobular structure in Fig 4C. (AVI)
