# Supplementary material for: Perturbation-Expression Analysis Identifies RUNX1 as a Regulator of Human Mammary Stem Cell Differentiation
Source: PLoS Comput Biol. 2015 Apr 20;11(4):e1004161. doi: 10.1371/journal.pcbi.1004161 (PMC4404314; doi:10.1371/journal.pcbi.1004161)
Supplement: S1 Text — (DOCX) [file pcbi.1004161.s001.docx]

Supplemental Experimental Procedures:

**IF Analysis of MCF10A Organoids**

Collagen culture pads were fixed with 4% paraformaldehyde for 15 minutes at room temperature. Pads were permeabilized using 0.1% TritonX-100 and incubated with blocking solution (PBST with 10% goat serum and 3% BSA) for 1 hr at room temperature. The following primary antibodies were used according to the manufacturer’s instructions: CK14 (Thermo Scientific-RB-9020-P), CK8/18 (Vector Labs-VP-C407), MUC1 (Abcam-ab-15481), CSN2 (Santa Cruz-sc-30041). An Alexa Fluor-labeled secondary antibody was used accordingly. Images were acquired using Zeiss700 confocal microscope.

**Clonal Analysis of MCF10A Organoids**

MCF10A cells were infected with viruses from LeGO lentiviral vectors kindly provided by Kristoffer Riecken (Weber et al., 2011). Virus was produced from three separate vectors encoding mCherry, Venus, and Cerulean fluorescent proteins. MCF10A cells were infected with all three viruses at a multiplicity of infection of approximately 0.25, as previously described. These cells were placed in collagen culture as previously described. Images were acquired after 2 and 6 days at 10X magnification using a Zeiss Axiovert 25 inverted microscope.

Supplemental Citations:

Weber, K., Thomaschewski, M., Warlich, M., Volz, T., Cornils, K., Niebuhr, B., Täger, M., Lütgehetmann, M., Pollok, J.-M., Stocking, C., et al. (2011). RGB marking facilitates multicolor clonal cell tracking. Nat. Med. *17*, 504–509.
