# Supplementary material for: Perturbation-Expression Analysis Identifies RUNX1 as a Regulator of Human Mammary Stem Cell Differentiation
Source: PLoS Comput Biol. 2015 Apr 20;11(4):e1004161. doi: 10.1371/journal.pcbi.1004161 (PMC4404314; doi:10.1371/journal.pcbi.1004161)
Supplement: S1 Table — (DOCX) [file pcbi.1004161.s003.docx]

Supplemental Table 1.

| FOXO1 | STAT1 |
| --- | --- |
| HOXA5 | STAT5A |
| LEF1 | XBP1 |
| GR | SP1 |
| GATA3 | HIF1A |
| SLUG | SREBF1 |
| EGR1 | PBX1 |
| CEBPA | E2F4 |
| ARNT | GAPDH |
