# Supplementary material for: Perturbation-Expression Analysis Identifies RUNX1 as a Regulator of Human Mammary Stem Cell Differentiation
Source: PLoS Comput Biol. 2015 Apr 20;11(4):e1004161. doi: 10.1371/journal.pcbi.1004161 (PMC4404314; doi:10.1371/journal.pcbi.1004161)
Supplement: S2 Table — (DOCX) [file pcbi.1004161.s004.docx]

Supplemental Table 2.

| SLUG | SREBF1 | POU2F1 (OCT 1) |
| --- | --- | --- |
| SOX9 | SRF | USF1 |
| NR3C1 (GR) | PBX1 | NF1 |
| GATA3 | HOXA5 | STAT5A |
| MSX1 | TCF3 | XBP1 |
| MSX2 | MAF | EGR1 |
| LEF1 | SP1 | NFYA |
| SOX5 | HIF1A | BPTF |
| RUNX1 | NFE2L1 | ETS1 |
| RB1 | AHR | ARNT |
| FOX01 | MYB | STAT1 |
| CEBPB | GABPA | YY1 |
| RBP-J | CEBPA | E2F4 |
