# Supplementary material for: Perturbation-Expression Analysis Identifies RUNX1 as a Regulator of Human Mammary Stem Cell Differentiation
Source: PLoS Comput Biol. 2015 Apr 20;11(4):e1004161. doi: 10.1371/journal.pcbi.1004161 (PMC4404314; doi:10.1371/journal.pcbi.1004161)
Supplement: S3 Table — Displayed are the PEACS scores, uncorrected p-value, Bonferroni corrected p-value, and significance (* = raw p<0.01; † = Bonferroni-corrected p<0.05) for genes with at least 3 knockdown conditions with 2-fold or higher knockdown. The negative control sets were generated by taking three random sets of 5 hairpins where the targeted gene was not successfully knocked down. P-values were obtained through Monte Carlo resampling of the PEACS scores, as described in the text. (DOCX) [file pcbi.1004161.s005.docx]

Supplemental Table 3.

| **Gene** | **PEACS Score** | **P-Value** | **Bonf Corr P** | **Significance** |
| --- | --- | --- | --- | --- |
| NR3C1 | 4.75 | <0.0001 | <0.0001 | *† |
| RUNX1 | 3.29 | 0.0003 | 0.0081 | *† |
| TCF3 | 3.01 | 0.0020 | 0.0540 | * |
| NF1 | 2.68 | 0.0035 | 0.0945 | * |
| RBPJ | 2.63 | 0.0036 | 0.0972 | * |
| AHR | 2.04 | 0.0327 | 0.8829 | * |
| RB1 | 2.01 | 0.0361 | 0.9747 | * |
| SRF | 1.92 | 0.0624 | 1.6848 |  |
| XBP1 | 1.87 | 0.0567 | 1.5309 |  |
| NFE2L1 | 1.71 | 0.1029 | 2.7783 |  |
| SOX5 | 1.53 | 0.1875 | 5.0625 |  |
| ETS1 | 1.46 | 0.1820 | 4.9140 |  |
| STAT1 | 1.39 | 0.2392 | 6.4584 |  |
| LEF1 | 1.21 | 0.3756 | 10.1412 |  |
| PBX1 | 1.13 | 0.3861 | 10.4247 |  |
| USF1 | 1.10 | 0.4077 | 11.0079 |  |
| HIF1A | 0.99 | 0.5201 | 14.0427 |  |
| SREBF1 | 0.96 | 0.5259 | 14.1993 |  |
| STAT5a | 0.84 | 0.6723 | 18.1521 |  |
| MSX2 | 0.83 | 0.6807 | 18.3789 |  |
| GABPA | 0.82 | 0.7197 | 19.4319 |  |
| E2F4 | 0.73 | 0.7417 | 20.0259 |  |
| SP1 | 0.66 | 0.8027 | 21.6729 |  |
| MSX1 | 0.60 | 0.8264 | 22.3128 |  |
| GATA3 | 0.59 | 0.8506 | 22.9662 |  |
| MAF | 0.58 | 0.8680 | 23.4360 |  |
| MYB | 0.51 | 0.9163 | 24.7401 |  |
| NEG 1 | 1.16 | 0.3716 | 10.0332 |  |
| NEG 2 | 0.95 | 0.5544 | 14.9688 |  |
| NEG 3 | 1.28 | 0.2886 | 7.7922 |  |
