# Supplementary figures and images for: Perturbation-Expression Analysis Identifies RUNX1 as a Regulator of Human Mammary Stem Cell Differentiation
Source: PLoS Comput Biol. 2015 Apr 20;11(4):e1004161. doi: 10.1371/journal.pcbi.1004161 (PMC4404314; doi:10.1371/journal.pcbi.1004161)

Supplemental Figure 1.


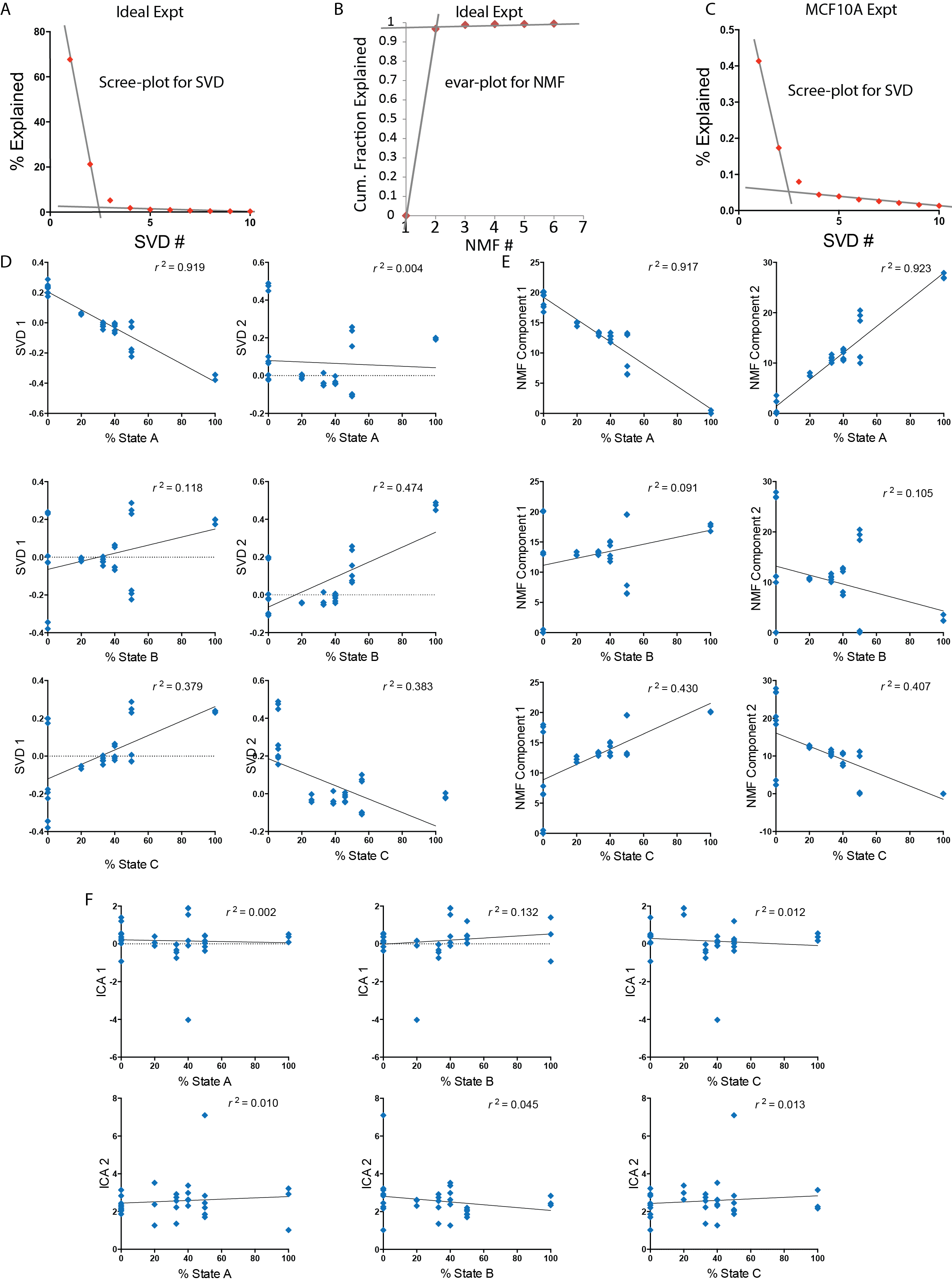

Supplement: S1 Fig — Scree plots and explained variance plots were used to decide on dimensions for SVD and NMF, respectively. These results are displayed as scatter plots where (A) the x-axis contains the SVD number and the y-axis denotes the variance explained by each SVD in the ideal experiment or (B) the x-axis contains the rank used by the NMF algorithm and the y-axis shows the fraction explained by all the components of the factorization in the ideal experiment. Similarly, (C) A scree plot of the SVD results from the MCF10A experiment was plotted to decide on dimensionality, where axes are as noted in (A). The results of first and second dimensions of (D) SVD, (E) NMF, and (F) ICA deconvolution were plotted against fractions of state A, B, or C. (DOCX) [file pcbi.1004161.s006.docx]

Supplemental Figure 2.


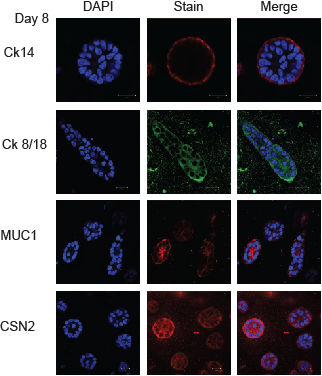

Supplement: S2 Fig — Day 8 collagen cultures were stained for basal marker (CK14) and luminal markers (CK8/18, MUC1 and CSN2). Nuclei were stained with DAPI. Scale bar, 20 μm. (DOCX) [file pcbi.1004161.s007.docx]

Supplemental Figure 3.


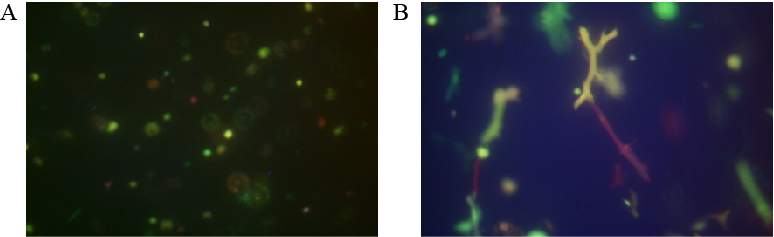
­­­­

Supplement: S3 Fig — MCF10A cells infected with a pool of red, green, and blue viruses were seeded into collagen matrix. The structures were visualized in the red, green, and blue channel (overlay shown) at 2 (A) and 6 days (B), revealing monoclonal lobules and monoclonal ducts with occasional fusions. Images were acquired at 10X magnification. (DOCX) [file pcbi.1004161.s008.docx]
